# Rapid Identification of Key Copy Number Alterations in B- and T-Cell Acute Lymphoblastic Leukemia by Digital Multiplex Ligation-Dependent Probe Amplification

**DOI:** 10.3389/fonc.2019.00871

**Published:** 2019-09-13

**Authors:** Deepshi Thakral, Gurvinder Kaur, Ritu Gupta, Anne Benard-Slagter, Suvi Savola, Indresh Kumar, Rajni Anand, Lata Rani, Pramod Verma, Sangeeta Joshi, Lalit Kumar, Atul Sharma, Sameer Bakhshi, Rachna Seth, Vivek Singh

**Affiliations:** ^1^Laboratory Oncology Unit, Dr. BRA IRCH, All India Institute of Medical Sciences, New Delhi, India; ^2^MRC Holland, Department of Tumour Diagnostics, Amsterdam, Netherlands; ^3^Department of Medical Oncology, Dr. BRA IRCH, All India Institute of Medical Sciences, New Delhi, India; ^4^Department of Pediatrics, All India Institute of Medical Sciences, New Delhi, India

**Keywords:** copy number alterations, CNAs, B-cell acute lymphoblastic leukemia, T-cell acute lymphoblastic leukemia, digital multiplex ligation-dependent probe amplification, dMLPA, MLPA, MRD

## Abstract

Recurrent clonal genetic alterations are the hallmark of Acute Lymphoblastic Leukemia (ALL) and govern the risk stratification, response to treatment and clinical outcome. In this retrospective study conducted on ALL patient samples, the purpose was to estimate the copy number alterations (CNAs) in ALL by digitalMLPA (dMLPA), validation of the dMLPA data by conventional MLPA and RT-PCR, and correlation of CNAs with Minimal Residual Disease (MRD) status. The ALL patient samples (*n* = 151; B-ALL, *n* = 124 cases and T-ALL, *n* = 27 cases) were assessed for CNAs by dMLPA for detection of sub-microscopic CNAs and ploidy status. This assay allowed detection of ploidy changes and CNAs by multiplexing of karyotyping probes and probes covering 54 key gene targets implicated in ALL. Using the dMLPA assay, CNAs were detected in ~89% (*n* = 131) of the cases with 66% of the cases harboring ≥3 CNAs. Deletions in *CDKN2A/B, IKZF1*, and *PAX5* genes were detectable in a quarter of these cases. Heterozygous and homozygous gene deletions, and duplications were observed in genes involved in cell cycle control, tumor suppression, lineage differentiation, lymphoid signaling, and transcriptional regulators with implications in treatment response and survival outcome. Distinct CNAs profiles were evident in B-ALL and T-ALL cases. Additionally, the dMLPA assay could reliably identify ploidy status and copy number-based gene fusions (*SIL-TAL1, NUP214-ABL, EBF1-PDGFRB*). Cases of B-ALL with no detectable recurrent genetic abnormalities could potentially be risk stratified based on the CNA profile. In addition to the commonly used gene deletions for risk assessment (*IKZF1, EBF1, CDKN2A/B*), we identified a broader spectrum of gene alterations (gains of- *RUNX1, LEF1, NR3C2, PAR1, PHF6;* deletions of- *NF1, SUZ12, MTAP*) that significantly correlated with the status of MRD clearance. The CNAs detected by dMLPA were validated by conventional MLPA and showed high concordance (*r* = 0.99). Our results demonstrated dMLPA to be a robust and reliable alternative for rapid detection of key CNAs in newly diagnosed ALL patients. Integration of ploidy status and CNAs detected by dMLPA with cytogenetic and clinical risk factors holds great potential in further refinement of patient risk stratification and response to treatment in ALL.

## Introduction

Acute lymphoblastic leukemia (ALL), the most common childhood cancer manifests as a clinically and genetically heterogenous malignancy. Both B-cell and T-cell ALL are primarily initiated by recurrent chromosomal translocations and driven by accumulation of distinct constellations of gross and sub-microscopic somatic genetic alterations including copy number alterations (CNAs), aneuploidy, structural variants, and DNA sequence mutations (1–4). Several of these lesions are important determinants of the risk of treatment failure and disease relapse but a universal agreement for inclusion of these genetic alterations in clinical risk stratification is still lacking. With current treatment protocols, long-term survival approaches 90%; however, a fraction of patients with favorable genetics relapse and refractory disease still poses a significant clinical challenge due to resistance of the recurrent disease to chemotherapy. Therefore, a more robust comprehensive genetic profiling can potentially facilitate better risk stratification and appropriate therapy.

The specific genetic subtypes in B-ALL are associated with distinct prognostic outcomes e.g., t(9;22) *BCR-ABL1* translocation, MLL gene rearrangements, hypodiploidy, and intrachromosomal amplification of chromosome 21 (*iAMP21*) predict poor outcome and genetic hallmarks such as *ETV6-RUNX1* and hyperdiploid ALL predict favorable outcome (1, 5). An integrated cytogenetic and molecular genetics approach has been proposed and validated in small cohorts for improved risk stratification in B-ALL and classification of patients into clinically relevant subtypes with potentially important implications for therapy (6–9). In addition to gross chromosomal rearrangements, partial, or complete deletions of the *IKZF1, CDKN2A/B, EBF1*, and *RB1* genes have emerged as independent predictors of poor outcome in B-ALL (10–12).

In the past two decades, genomic-profiling approaches have provided important insights into the genetic basis of ALL. ALL harbors several recurring regions of genetic alterations, most commonly focal submicroscopic or cryptic lesions that are not evident on conventional cytogenetic analysis (13). Many of the genes altered in ALL encode proteins with roles in key cellular pathways, including lymphoid development and differentiation (e.g., *PAX5, IKZF1, EBF1, LEF1*, and *VPREB*), cell-cycle regulation and tumor suppression [*CDKN2A, CDKN2B (INK4/ARF), TP53, PTEN*, and *RB1*], lymphoid signaling (*BTLA, CD200*, and *TOX*), transcription factors and transcriptional coregulators (*ERG, TBL1XR1*, and *CREBBP*), regulation of apoptosis (*BTG1*), and drug receptor (*NR3C1*) (10). Importantly, studies have also identified genetic lesions that define novel subtypes of ALL with distinct gene expression profiles, such as subtypes characterized by rearrangement of *CRLF2* (14, 15), intrachromosomal amplification of chromosome 21 (16), focal deletions of *ERG* (17), and *NOTCH1* (18). Several of these alterations described are clinically relevant in terms of risk stratification of ALL patients.

Recently, a next-generation sequencing-based Multiplex Ligation-dependent Probe Amplification (MLPA) variant, digitalMLPA (dMLPA) was developed to detect sub-microscopic CNAs, which might be missed by existing conventional, and high-resolution technologies (19). The available molecular diagnostic tools for detection of these key genetic abnormalities to distinguish various subtypes of ALL include conventional karyotyping, FISH, aCGH, SNP arrays, and conventional MLPA which are tedious and time-consuming. dMLPA allows multiplexing of several targets for accurate copy number detection of multiple genomic targets implicated in ALL. The aim of our study was to evaluate the utility of dMLPA in screening of ALL patients for key CNAs. Here, we present (1) the spectrum of chromosomal abnormalities, and CNAs including gene alterations among B-ALL and T-ALL patients, (2) validation of dMLPA data by conventional MLPA and RT-PCR, (3) correlation of CNAs with Minimal Residual Disease (MRD) status and integration of CNAs with risk stratification in ALL. As far as we know, our study demonstrated the utility of dMLPA in the detection of CNAs in ALL for the first time in Indian patients.

## Materials and Methods

### Patients

In this retrospective study, 151 consecutive samples of ALL patients registered at Dr. BRA IRCH, All India Institute of Medical Sciences, New Delhi, India from June 2017 to November 2018 were included. The diagnosis of ALL was made as per the updated criteria of WHO classification of myeloid neoplasms and acute leukemia (20). Waiver for informed consent was obtained from the Institute Ethics Committee (IEC-573/03.11.2017).

### Risk Stratification and Treatment Regimen

The patients were risk stratified as standard (age between 1 and 10 years, TLC < 50 × 10^9^/L, absence of high risk cytogenetics and CNS involvement and good prednisolone response on day 8 post induction chemotherapy i.e., blasts in peripheral blood <1 × 10^9^/L), intermediate (age>10 years/ TLC > 50 × 10^9^/L/ organ enlargement and absence of any high risk cytogenetic) and high risk groups (high risk cytogenetics, CNS involvement, poor early prednisolone response, and MRD positivity post induction).

The treatment protocol used for the pediatric patients (below 18 years) was according to the Indian Childhood Collaborative Leukemia (ICICLE; Clinical Trials Registry-India Reference Number, unpublished CTRI/2015/12/006434) Group protocol. Briefly, the induction regimen for standard risk group consisted of three chemotherapeutic agents (prednisolone, vincristine, and L-asparaginase). Daunomycin was added as the fourth drug for intermediate to high risk patients. The follow up of these patients depended on MRD status and the treatment included consolidation, maintenance, delayed intensification, and maintenance chemotherapy for two years. The adult patients were treated according to the MCP-841 protocol (21). Patients who failed to achieve complete remission or relapsed were given re-induction therapy according to the UK-ALL XII protocol (22). Patients positive for BCR-ABL additionally received *Imatinib mesylate* (>1 year 340 mg/m^2^/day; not exceeding 600 mg/day).

The MRD status was assessed at the end of induction chemotherapy using 10-color multi-parameter flow cytometry (Gallios, Beckman Coulter, Brea, CA). Stain-lyse-wash method was used with fluorochrome-tagged antibodies namely, CD81, CD45, CD34, CD58, CD19, HLA-DR, CD10, CD20, CD123, CD38 (Beckman Coulter, Miami, FL, USA). At least, one million cells were acquired in each case and list mode files were analyzed by Kaluza software version 3.0 (Beckman Coulter, Brea, CA, USA).

### RT-PCR for Detection of Balanced Translocations

RNA was isolated using Tri Reagent (SIGMA-ALDRICH) as per the manufacturer's instructions. First strand cDNA was generated using RevertAid First Strand cDNA Synthesis Kit (Thermo Scientific, Waltham, Massachusetts, United States) as per the manufacturer's instructions. The product of the first strand cDNA synthesis was used directly in PCR for the detection of fusion transcripts including *BCR-ABL1, ETV6-RUNX1, KMT2A-AFF1, E2A-PBX1*, and *STIL-TAL1* (23).

### DigitalMLPA for Detection of Copy Number Alterations and Ploidy Status

DNA was isolated from either bone marrow or peripheral blood collected at the time of diagnosis using Genomic DNA Purification Kit (GeneJET, ThermoFisher Scientific, Waltham, Massachusetts, United States). For performing digitalMLPA, the modified well-established MLPA protocol was combined with Illumina next generation sequencing Miseq platform for amplicon quantification as described earlier (19). Probes for detection of both B-ALL and T-ALL associated Copy Number Alterations (CNAs) were included in this digitalMLPA D007 ALL probemix, which can multiplex >600 probes in a single reaction and simultaneously perform copy number analysis of 54 key target genes. In addition, 198 probes in the same probemix were included for karyotyping and data normalization purposes and a set of 128 control probes were included for quality control purposes.

Briefly, 100 ng of each DNA sample (in a total volume of 4 μl) sample was mixed with 2 μl of a unique barcode solution (MRC Holland), followed by DNA denaturation at 98°C for 10 min. After denaturation, a mixture of 1.25 μl dMLPA probe mix (MRC Holland) and 1.25 μl dMLPA buffer (MRC Holland) was added to each sample, and reactions were incubated overnight at 60°C for hybridization. Probes were ligated by incubating the reactions with 32 μl of a ligase master mix containing the ligase-65 enzyme (MRC Holland) and buffers (MRC Holland) at 48°C for 30 min, followed by heat inactivation of the ligase-65 enzyme at 98°C for 5 min and an additional incubation at 65°C for 20 min. PCR amplification of the ligated probes was performed on a calibrated Veriti 96-well thermocycler (Applied Biosystems). The PCR-amplified products were pooled and the library was loaded onto an Illumina MiSeq sequencer (Illumina, San Diego, CA) for quantification using the MiSeq Reagent Kit version 3 (150 cycles; Illumina). Sixteen healthy control DNA samples were included in the first validation run and for subsequent runs eight reference controls were included. Relative peak ratios between 0.8 and 1.2 were considered normal, while values below or above indicated losses or gains of genetic material, respectively. Data analysis was performed as described previously (19). Finally, heatmaps were generated comparing each dMLPA probe in each test sample against that in a reference sample to determine copy number changes for the indicated gene target.

### Validation by Conventional MLPA

Conventional MLPA experiments were performed with DNA isolated from bone marrow or peripheral blood samples. Several SALSA MLPA probemixes were used for CNAs identification in these genes (MRC Holland, Amsterdam, The Netherlands). These probemixes included probes for *IKZF1, CDKN2A/B, PAX5, EBF1, ETV6, BTG1, RB1*, and *PAR1* region (P335 & P202 kit), iAMP21-ERG (P327 probemixes) and a probe mix for T-ALL (P383) containing several different targets. The results of MLPA analysis were interpreted by assigning deletion status to the indicated genes for each sample. For each probe the status was assigned as “deleted,” “normal,” or “amplified” depending on the ratio obtained relative to the established normal range (mean ± 2SD or mean ± 3SD). Relation dosage quotient values below 0.8 were considered deletion and above 1.2 as amplification. If more than 50% of the probe ratios in a particular region indicated a copy number alteration of the genetic material, the result for that gene was designated abnormal. Deletion of either *CDKN2A* or *CDKN2B* was considered as *CDKN2A/B* locus to be deleted. For deletion in the *PAR1* region of chromosome X or Y, we have considered it synonymous with *P2RY8-CRLF2* as described earlier (6).

### Statistical Analysis

The significance of any change in DNA copy number for MLPA, and data from dMLPA, were compared using Pearson's correlation test as appropriate. All analyses were performed using SIGMAPLOT version 13.0 (SYSTAT Software, Inc. USA). Chi-square test and Fisher's exact test were used to correlate various parameters with the cytogenetic profile and CNAs. *p* < 0.05 were considered statistically significant. The FDR values and heat map were derived using R statistical software.

## Results

**Figure 1:**
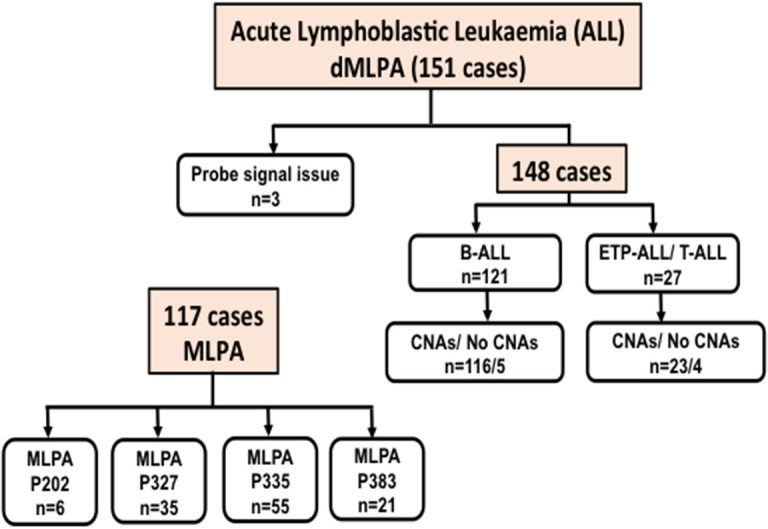
Flow-chart showing the number of Acute Lymphoid Leukemia (ALL) cases including B-cell and T-cell ALL analyzed for Copy Number Alterations (CNAs) by digitalMLPA and conventional MLPA. P202, P327, P335, and P383 indicate the conventional MLPA probemixes used and the number of cases analyzed.

In ALL samples, an average of 900 single reads of 100 nucleotides was generated with minimal variability relative to reference samples that showed a standard deviation below 0.1 (maximum 0.06) across all target probes. Of the 151 ALL samples, three were excluded from the analysis as high variability in read ratios was observed (Figure 1). This could be attributed to the presence of impurities in the DNA samples. The characteristics of ALL patients including gender, age, total leukocyte counts, laboratory parameters (*n* = 148), and clinical features (*n* = 92) are mentioned in Table 1.

**Table 1:** Baseline characteristics of ALL patients evaluated by dMLPA.

**Characteristics**	**Number (%)**
Total	148
**GENDER**	
Male	109 (73.6)
Female	39 (26.4)
**AGE RANGE (YEARS)**	
≤1	9 (6)
2–10	83 (56)
10–18	41 (28)
>18	15 (10)
**TLC**, **×10**^**9**^**/L**	
Median	144 (17.2)
Range	0.06–533.6
≤50	99 (68.75)
>50	45 (31.25)
**PERCENT BLAST**	
Median	85
**IMMUNOPHENOTYPE**	
Pro-B ALL	3
Common-ALL	103
Pre-B ALL	1
T-ALL	23
ETP-ALL	4
**KARYOTYPE (B-ALL)**	51/121
**MRD STATUS (B-ALL;** ***n*** **=** **92)**	
MRD positive	25
MRD negative	67

### Distribution Frequency of CNAs in ALL

**Figure 2:**
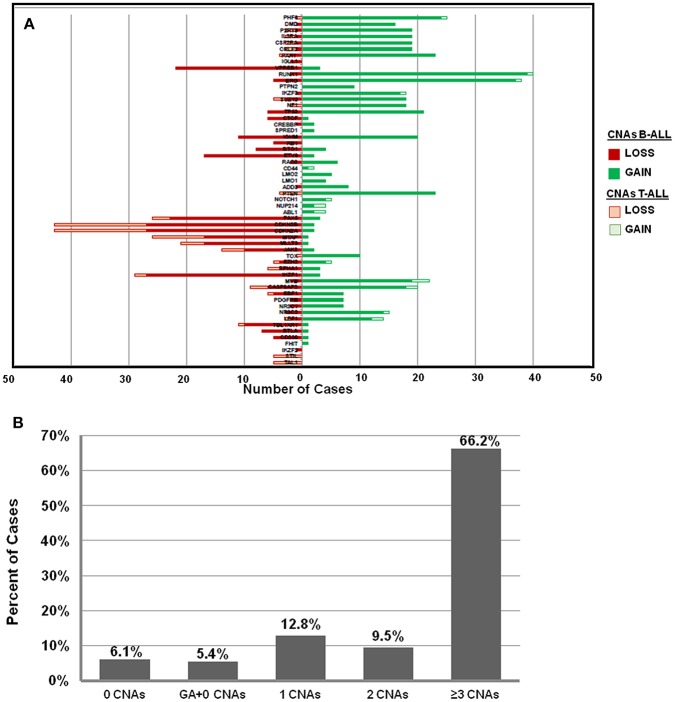
Frequency distribution of CNAs in ALL cases analyzed by dMLPA. **(A)** Gene deletions detected in samples of B-ALL (filled red bar) and T-ALL (Empty red bar) are shown in the left panel and gene duplications (filled and empty green bars, respectively) in the right panel. **(B)** Bar diagram showing percentage of cases with 0, 1, 2, and ≥3 CNAs, GA represents recurrent genetic chromosomal abnormalities.

The frequency distribution of CNAs detected in B-ALL (*n* = 121) were predominantly heterozygous or homozygous deletions in *IKZF1* (22%) and *CDKN2A/B* (22%) followed by *PAX5* (19%) (Figure 2A, Supplementary Table S1). Deletions were also detected in *MLLT3* (14%)*, MTAP* (14%)*, ETV6* (14%), and deletions in other gene targets were observed in <10% of the B-ALL cases. Gene duplications were observed in *RUNX1* (~32% cases), *ERG* (~30% cases), *PHF6* (20%), and *PTEN* (19%) genes and the following genes showed only duplication and no deletion was noted in any patient sample- *TOX, ABL1, NUP214, NOTCH, PTEN, LMO1, LMO2, CD44, SPRED1, NF1, PTPN2*, and *PHF6* genes (Figure 2A). In the 27 T-ALL cases (18% of total ALL cases), deletions were noted in 59% (*n* = 16) cases primarily in the *CDKN2A/B* gene locus followed by *MTAP* (~33%), 15% in *MLLT3/ PTEN/ JAK2*, and <10% in *PAR1, IKZF1, EZH2, EBF1*, and *PHF6* (Figure 2A; Supplementary Table S1).

Of the 148 ALL samples, no CNAs were detected in 9 samples (~6%) [B-ALL (*n* = 5), T-ALL (*n* = 2), and ETP-ALL (*n* = 2)]. Noticeably, 66% of the cases showed ≥3 CNAs, ~10% had 2 CNAs and ~13% had only 1 CNA (Figure 2B). Interestingly, in ~5% (*n* = 8 cases) of the samples, which harbored recurrent chromosomal abnormalities including *BCR-ABL1* (*n* = 1), *KMT2A-AFF1* (*n* = 2), and *E2A-PBX* (*n* = 5), no CNAs were detected. Copy number alterations identified were on an average of 5.6 somatic CNAs per sample (5.8 in B-cell precursor ALL and 3.8 in T-cell ALL) for the 54 gene targets covered.

### Spectrum of CNAs and Prognostic Significance in B-ALL

**Figure 3:**
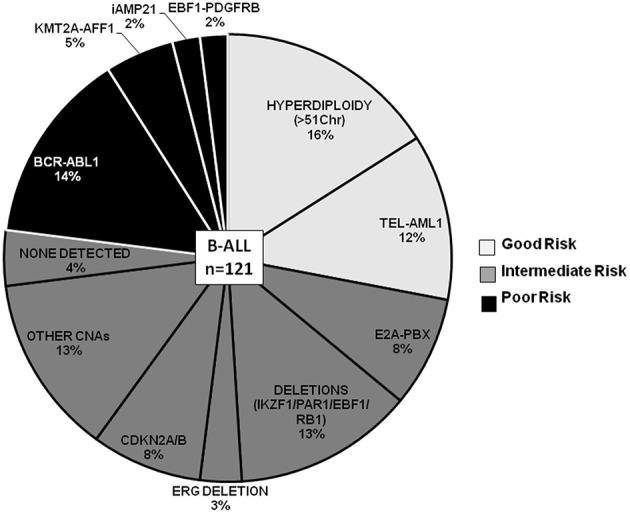
B-ALL cases categorized by recurrent cytogenetic abnormalities and CNAs. Pie chart depicting the frequency distribution of B-ALL cases with chromosomal abnormalities and CNAs. The various categories are distributed by risk groups and are color-coded as good risk (light gray), intermediate risk (dark gray), and poor risk (black).

For risk evaluation, B-ALL cases (~82% of total ALL cases) were categorized by cytogenetic risk groups as patients with good-risk (GR) genetic abnormalities (*n* = 33, ~27%), poor-risk (PR) abnormalities (*n* = 25; ~21%), and the remaining 52% (*n* = 63) of the cases as intermediate cytogenetic risk (IR) group (Figure 3). In the category of Good Risk (GR) genetic abnormalities (High hyperdiploidy and *ETV6-RUNX1*), 16% (*n* = 19) of the B-ALL cases were assigned Hyperdiploidy status as determined by the karyotyping probes in the dMLPA assay. Only chromosomes for which all karyotyping probes showed increased read ratios of greater than the cut-off of 1.3 and above were considered as heterozygous gain or >1.75 as homozygous gain whereas partial chromosomal gain were excluded. Concordance of hyperdiploidy status could be demonstrated in three cases where simultaneous conventional karyotyping data was available (Supplementary Table S2). Non-random gain of chromosomes X, 4, 6, 10, 14, 17, 18, and 21 was commonly observed with exceptions of gain of chromosomes 1, 5, 7, and 8 in a few cases (Supplementary Figure S1). In cases with hyperdiploidy, isolated CNAs were noted as deletions in *CDKN2A/B, MTAP, IKZF1, CASP8AP2, ERG, RAG2, ETV6, CTCF, TBL1XR1*, and *VPREB1* genes, which could potentially introduce heterogeneity for risk stratification in this good risk category (Supplementary Table S2).

**Figure 4:**
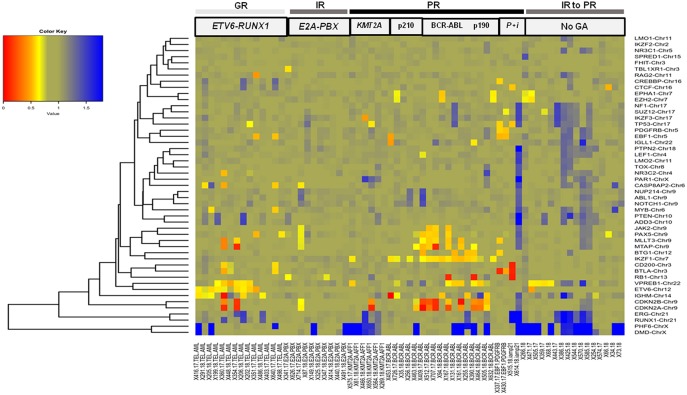
B-ALL cases categorized by cytogenetic abnormalities showing heterogeneous distribution of CNAs. Heat map showing recurrent chromosomal abnormalities in Good risk (GR) category included *ETV6-RUNX1* (hyperdiploidy cases not shown), Intermediate risk (IR) represented by *E2A-PBX* (samples with *IKZF1, EBF1, RB1, ERG*, and *CDKN2A/B* deletions not shown) and Poor risk (PR) included *KMT2A-AFF1, BCR-ABL1* (cases with p210 and p190 isoforms), *EBF1-PDGFRB* (indicated by P), and *iAMP21* (indicated by i). Also included in this heat map are CNAs in B-ALL cases with no detectable cytogenetic abnormalities (indicated by No GA). (The color key shows a gradient from red to blue wherein red indicates homozygous deletion, yellow and orange indicate heterozygous deletion and blue indicates duplication. The gene names are labeled on the right hand side and the patient sample IDs with or without genetic abnormality are indicated in the bottom row).

The other GR category comprised 12% (*n* = 14 cases) of the B-ALL cases with *ETV6-RUNX1* genetic abnormality (Figure 3), wherein predominant CNAs were observed as gene deletions in the *ETV6* gene indicating rearrangements and fusions (Figure 4). In addition, deletions were also observed in *PAX5* (3 cases), *IGHM* (6 cases), *VPREB1* (6 cases), *EBF1*, and *MYB* (2 cases) genes. No case with *ETV6-RUNX1* harbored deletions in *IKZF1, PAR1*, or *BTG1*. An isolated case within this category showed a deletion in the *RB1* gene. Two cases with a deletion in the *EBF1* gene showed co-occurrence of deletions in *NR3C2, PAX5, RAG2*, and *VPREB1* or deletions in multiple other genes (*CD200, BTLA, CASP8AP2*, and *MYB*). Also, gene duplications were detected in *RUNX1, ERG, PHF6*, and *PTEN* genes in a few cases (Figure 4).

In the poor risk (PR) category, the dominant CNAs detected in B-ALL with *BCR-ABL* genetic abnormalities **(**14%; *n* = 17 cases) (Figure 3) were deletions in *IKZF1* (~82%; *n* = 14 cases) and *CDKN2A/B* locus (~53%; *n* = 9 cases) (Figure 4). Majority of the cases showed partial or complete deletion of the exonic region of *IKZF1* gene but in one case heterozygous deletion in the genomic region immediately upstream of *IKZF1* gene was noted. Mutually exclusive deletions in *BTG1, IGHM, PAX5*, and *VPREB1* genes were also present in almost 25% of the cases. The *BCR-ABL* isoform (p210) was detected in the five samples and the remaining 12 samples showed the p190 isoform. Interestingly, the CNA profile observed in the cases with p190 isoform were more heterogenous as compared to the cases with p210; the latter harbored only *IKZF1* or *CDKN2A/B* gene deletions (Figure 4). One case also harbored a rare *NUP214-ABL1* intrachromosomal rearrangement, a rare entity seen in B-ALL.

In the other poor risk (PR) category of B-ALL with *KMT2A-AFF1* (5%; *n* = 6 cases) genetic abnormalities (Figure 3), gene deletions were detected in *IKZF1, CDKN2A/B, TP53, IKZF3*, and *VPREB1* genes. No gene deletions were detected in three cases. Interestingly, four cases harbored gene duplications in *DMD* and *PHF6* genes (Figure 4). One case of B-ALL with iAMP21 amplification showed gene deletions in *RB1, TP53, CTCF, CD200, and BTLA* (Figure 4; Supplementary Figure S2). Intrachromosomal *EBF1-PDGFRB* rearrangement was detected by dMLPA in two cases, which also carried deletions in *SUZ12, BTG1, BTLA*, and *TP53* genes associated with poor risk (Figure 4; Supplementary Figure S2).

Contrary to the GR category with *ETV6-RUNX1* genetic abnormality, cases in the intermediate risk category with *E2A-PBX* (8%; *n* = 10 cases) (Figure 3) chromosomal abnormality rarely showed any gene deletions. Three out of ten cases showed deletions in *RB1* and *PAX5* genes with co-occurrence of *TBL1XR1* or *BTG1* or *CDKN2A/B, CASP8AP2*, and *JAK2* gene deletions (Figure 4).

The remaining ~42% cases with no detectable cytogenetic abnormalities were classified as intermediate-risk group and further evaluated based on CNAs (loss or gain) detected by dMLPA. Of these, 16% (*n* = 20 cases) showed deletions in *IKZF1/PAR1/EBF1/RB1/ERG*, and 8% (*n* = 10) of these cases carried deletions in *CDKN2A/B* locus, which are associated with poor risk (Figure 3). Within this subgroup, one case also harbored a *NUP214-ABL1* intrachromosomal rearrangement, a rare entity seen in B-ALL. The remaining 13% (*n* = 16 cases) displayed substantial heterogeneity in the CNAs detected (Figure 4). Strikingly, ~50% of these cases showed duplications in *RUNX1* and *ERG* and two cases in *DMD, MYB*, and *CASP8AP2*. Four cases harbored a deletion in the *VPREB1* gene, and two cases showed gene deletions in *EZH2* and *EPHA1*. Interestingly, intragenic amplification of exon 2–5 of the *PAX5* gene was detected in one patient sample that was also MRD positive.

The CNAs (both gene deletions and duplications) were correlated with post-induction MRD status, which was available for 92 cases of which 27% (*n* = 25) cases were MRD positive (≥0.01%) and the remaining 73% (*n* = 67) were MRD negative (Table 1). In addition to the previously described CNAs of prognostic relevance (*IKZF1, CDKN2A, BTG1, EBF1, PAX5, ETV6, RB1*), we observed a correlation between deletions in *NF1* and *SUZ12* and duplications in *LEF1, NR3C2, RUNX1*, and *PAR1* region and MRD positivity (Table 2). A trend of correlation was also seen for *PHF6* and *CASP8AP2* gene duplication and MRD positivity.

**Table 2:** Correlation of Minimal Residual Disease status with Deletions and Duplications in individual genes/loci.

**CNA in genes**	**MRD −**	**MRD+**	**Fisher's exact (*p*)**	**FDR (p. adj.)**	**CNA in Genes**	**MRD −**	**MRD+**	**Fisher's exact (*p*)**	**FDR (p. adj.)**
IKZF1 deletion	13	6	0.77	1	LEF1 gain	2	6	0.005^*^	**0.08**
IKZF wt	54	19			LEF1 wt	64	19		
CDKN2A/B deletion	18	2	**0.08**	0.7	NR3C2 gain	3	6	0.010^*^	**0.08**
CDKN2A/B wt	48	22			NR3C2 wt	62	18		
BTG1 Deletion	4	1	1	1	RUNX1 gain	14	12	0.015^*^	**0.08**
BTG1 wt	61	24			RUNX1 wt	50	11		
EBF1 deletion	3	0	0.55	0.95	PAR1 gain	10	9	0.042^*^	0.17
EBF1 wt	58	25			PAR1 wt	56	16		
PAX5 deletion	16	3	0.38	0.86	PHF6 gain	10	8	0.081	0.25
PAX5 wt	49	20			PHF6 wt	57	17		
ETV6 deletion	13	4	0.77	1	CASP8AP2 gain	6	6	0.092	0.25
ETV6 wt	53	21			CASP8AP2 wt	57	19		
Rb1 deletion	3	0	0.56	0.95	PTPN2 gain	2	3	0.122	0.28
Rb1 wt	64	25			PTPN2 wt	65	22		
MLLT3 deletion	12	2	0.33	0.86	MYB gain	7	6	0.177	0.35
MLLT3 wt	54	23			MYB wt	58	19		
MTAP deletion	16	2	0.14	0.79	PTEN gain	9	6	0.224	0.4
MTAP wt	50	23			PTEN wt	58	19		
NF1+SUZ12 del.	0	2	**0.06**	0.7	TOX gain	5	4	0.261	0.42
NF1+SUZ12 wt	61	19			TOX wt	59	21		
EPHA1 deletion	2	2	0.3	0.86	ADD3 gain	3	3	0.34	0.47
EPHA1 wt	64	23			ADD3 wt	63	22		
EZH2 deletion	2	2	0.3	0.86	IGHM gain	10	6	0.35	0.47
EZH2 wt	64	23			IGHM wt	49	16		
VPREB deletion	13	7	0.41	0.86	DMD gain	6	4	0.446	0.55
VPREB wt	53	18			DMD wt	61	20		
CD200/BTLA deletion	4	1	1	1	PDGFRB gain	3	0	0.5	0.57
CD200/BTLA wt	62	23			PDGFRB wt	64	25		
TBL1XR1 deletion	5	1	1	1	NR3C1 gain	4	0	0.57	0.61
TBL1XR1 wt	62	23			NR3C1 wt	62	24		
IGHM deletion	8	3	1	1	LMO1 gain	4	2	0.668	0.67
IGHM wt	49	16			LMO1 wt	61	23		

* *and bold p < 0.05 were considered statistically significant*.

*p values showing a trend of correlation are shown in bold*.

### Frequency Distribution and Prognostic Value of CNAs in T-ALL

**Figure 5:**
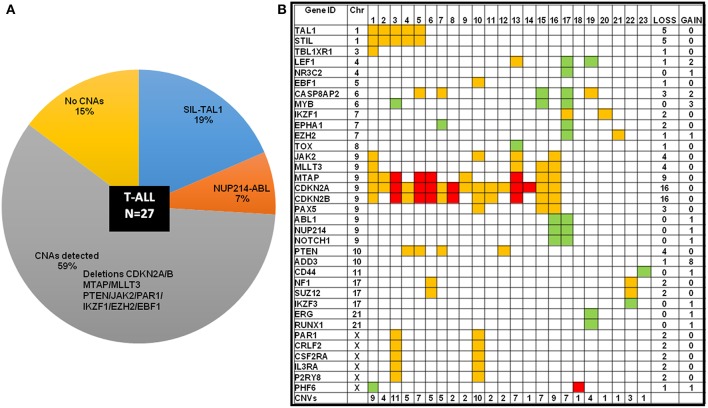
T-ALL cases categorized by cytogenetic abnormalities showing distribution of CNAs. **(A)** T-ALL cases sub-categorized by chromosomal abnormalities and CNAs in a pie-chart. **(B)** Heat map showing the CNAs distribution in T-ALL cases. The color codes indicate red as homozygous deletion, orange indicate heterozygous deletion and green indicates duplication.

Intrachromosomal fusions were detected in ~19% cases, with five cases showing a *SIL-TAL1* fusion and two cases showed *NUP214-ABL* genetic rearrangement (Figure 5A; Supplementary Figure S3). All cases with *SIL-TAL1* fusion and one case with *NUP214-ABL* also carried *CDKN2A/B* gene deletions (Figure 5B). Deletions in *CDKN2A/B, IKZF1*, and *EZH2* were mutually exclusive. Two cases showed *NF1/ SUZ12* deletions associated with poor risk. Four cases including two cases of ETP-ALL did not show any CNAs (not shown in the heat map Figure 5B).

### Validation of dMLPA by Conventional MLPA and RT-PCR

The CNAs detected by dMLPA were confirmed by conventional MLPA assays (P202, P335, P327, and P383) as per the availability of samples (*n* = 117). The reference range data showed a normal distribution in each case, and a narrow variation in the mean and SD in the reference samples (Mean ± 2SD or 99% CI, Mean ± 3SD or 95% CI; *p* = 0.05), dosage quotient ranging from 0.8 to 1.2 (representative read ratios, Supplementary Figure S4). The degree of concordance between the conventional MLPA and dMLPA assays for the detection of listed genes indicating the number of samples tested is shown in Supplementary Table S3. The Pearson's correlation coefficient was calculated to determine the efficiency of dMLPA and conventional MLPA and a positive correlation (*r* = 0.99) between the two assay methods was observed. Heterozygous deletion in *EZH2* gene was detected in three cases by conventional MLPA as opposed to one case by dMLPA.

Chromosomal abnormalities including *BCR-ABL1, KMT2A-AFF1, E2A-PBX*, and *ETV6-RUNX1* were detected by RT-PCR and a few positive cases were also confirmed by FISH (FISH data was available for the following number of cases *BCR-ABL1* = 12/17; *KMT2A-AFF1* = 5/6; *E2A-PBX* = 7/10; and *ETV6-RUNX1* = 9/14). Intrachromosomal gene fusion, *STIL-TAL1* detected in five T-ALL cases was also validated by RT-PCR and conventional MLPA (Supplementary Figure S4). Also, positive cases for *NUP214-ABL1* were confirmed by conventional MLPA (P383 probemix).

## Discussion

In this study, the potential of detection of Copy Number Alterations by dMLPA assay and its integration with cytogenetic profiling for an improved genetic risk stratification of ALL patients was evaluated. After categorization of patients into cytogenetic risk groups as determined by recurrent chromosomal abnormalities, CNA profiles obtained by dMLPA could further aid in refining the classification of risk groups and prediction of clinical outcome. The previous study by Benard-Slagter et al. (19) demonstrated dMLPA as a robust and reliable tool for genetic characterization of ALL patients. We found this to be the case as we were able to detect CNAs in ~89% of our cases with dMLPA assay as opposed to conventional MLPA where almost 50% of the cases harboring rare (<10% cases) yet relevant CNAs are missed as limited targets are covered and multiple assays have to be performed. Moreover, dMLPA could detect ≥3 CNAs in 66% of our cases whereas that reported by conventional MLPA were shown in only 10% of the cases (6). Our study provides a validation of dMLPA assay and takes the previous study by Benard-Slagter et al. (19) to the next level for its potential translation into routine diagnostics and genetic risk stratification of ALL.

CNAs detected by dMLPA were validated by available MLPA assays that showed high concordance between the two methods [as also shown by (19)]. The multiplexing of ~600 probes in a single reaction of dMLPA rarely resulted in false positive or false negative results as confirmed by conventional MLPA assays. Indeed, a wide range of genomic targets could be analyzed for CNAs with less hands-on time and easier data analysis. With a limit of detection of CNAs in samples carrying 25–30% of neoplastic cells (19), in two B-ALL cases the blast percent was borderline (20–30%), which could have resulted in failure to detect CNAs.

Distinct CNA profiles were evident in B-ALL and T-ALL with both mutually exclusive and co-occurring genes. The dominance pattern of key genes affected in B-ALL cases was similar to earlier studies from India (*IKZF1, CDKN2A/B, PAX5, ETV6*) (8, 9, 12) and different from that reported by other groups where deletions in *CDKN2A/B* and *ETV6* were dominant (6). The new gene targets such as *BTLA, CD200, VPREB1* which are associated with inferior event-free and overall survival; *DMD* with relapse, *TBL1XR1* and *TP53* with chemo-resistance; *EZH2*, PAR1 region, and *P2RY8-CRLF2* with poor prognosis; *CASP8AP2, NF1*, and *SUZ12* associated with poor response to induction therapy are included in this dMLPA panel and useful for further risk stratification in cytogenetically good risk B-ALL cases. In contrast, cases with T-ALL but not B-ALL harbored deletions in *PTEN, PHF6*, and *TOX* genes. Inactivation of *PTEN* frequently occurs in T-ALL and may be associated with chemotherapy resistance and poor prognosis. The CNA profiles obtained using the dMLPA assay were potentially relevant in facilitating risk stratification of cases without recurrent or detectable genetic abnormalities and classical genetic alterations in our cases.

In the cases with hyperdiploidy, isolated CNAs were noted as deletions in *CDKN2A/B, IKZF1, CASP8AP2, ERG, RAG2, ETV6, CTCF, TBL1XR1*, and *VPREB1* genes, which could potentially introduce heterogeneity in this good risk category. As described by Benard-Slagter et al. (19) a rare intragenic deletion of *ERG* exons 5–9 was detected in two of our cases and a single deletion in exon 5 was also noted. Few cases harbored partial chromosomal deletions in Chromosomes 7 and 9 but we did not detect any case of hypodiploidy. Absence of deletions in *CDKN2A/B, IKZF1, PAR1*, and *BTG1* in cases with *ETV6-RUNX1* genetic abnormality indicated certain genetic predisposition of the combination of gene targets, which might be affected with the primary genetic abnormality. Two cases with a deletion in the *EBF1* gene showed co-occurrence of deletions in *NR3C2, PAX5, RAG2*, and *VPREB1* or deletions in multiple other genes (*CD200, BTLA, CASP8AP2, MYB*) influencing the risk stratification of these good risk category. Unlike reported in the previous study by Benard-Slagter et al. (19), which showed the presence of homozygous deletion of *CD200* and heterozygous deletion of *BTLA*, we identified a case of ALL relapse with heterozygous deletion of *CD200* and a homozygous deletion of *BTLA*. One case of B-ALL with *iAMP21* amplification showed homozygous deletion of *CD200* and *BTLA* and gene deletions in *RB1, TP53, CTCF* as has been reported earlier (24). An isolated case of intragenic amplification of *PAX5* gene with MRD positive status may define a novel, relapse-prone subtype of B-cell precursor ALL with a poor outcome but this need to be validated in larger cohort.

B-ALL with t(9;22) BCR-ABL1 chromosomal translocation is epidemiologically more prevalent in adults (~25%) and accounts for 2–4% of cases in children. These patients belong to the high risk category depending on the age, WBC count and response to therapy. In the majority of childhood cases, a p190 BCR-ABL1 fusion transcript is observed and p210 is rare. The distinct CNA profile observed in patients with p190 and p210 isoforms could be further evaluated using dMLPA. The cases with p210 isoform primarily harbored deletions in *IKZF1* gene whereas samples with p190 isoform had *IKZF1* plus profile (*CDKN2A/B, PAX5*, and other genes) associated with MRD-dependent very-poor prognostic profile in BCP ALL.

Several other common gene deletions, such as *CDKN2A/2B, IKZF1, PAX5, ETV6, RB1*, and *BTG1* have also been described in ALL patients (6, 25). Deletions in the *IKZF1* gene have been associated with relapse and poor clinical outcome in several studies. In ~80% of pediatric and 60–90% of adult *BCR-ABL1* positive ALL cases, partial or complete gene deletions of *IKZF1* are detected. Eighty percent of the B-ALL with *BCR-ABL1* chromosomal abnormality showed *IKZF1* deletions in our cohort. Partial gene deletions of *IKZF1* frequently affect exons 4–7 and was seen in majority of our cases (*n* = 10), but a rare smaller deletion, affecting a region upstream of *IKZF1* gene was detected which was not reported by Benard-Slagter et al. (19). Also, deletions of *IKZF1* exon 4–8 associated with poor outcome although are rare but were seen in two of our samples in which this gene was affected. Earlier studies have reported the prognostic relevance of deletions in *IKZF1* and *EBF1* genes which are associated with MRD positivity (6, 9, 12). In our retrospective study, since *IKZF1* deletions were primarily detected in *BCR-ABL1* positive B-ALL cases, these cases were risk stratified into the poor risk category and were given intensive chemotherapy. This could have resulted in skewing of the correlation between *IKZF1* and other gene deletions and MRD clearance in our cases. Individual poor risk chromosomal abnormalities were too rare to reliably test for correlation.

We successfully detected intrachromosomal fusions with copy number imbalance (*SIL-TAL1, NUP214-ABL, EBF1-PDGFRB*) in a few cases, which could potentially benefit from treatment with tyrosine kinase inhibitors (26–28). The *NUP214-ABL1* fusion gene is demonstrated to be amplified as multiple (5–50) episomal copies in 6% of T-cell acute lymphoblastic leukemia (T-ALL) and has been reported as a rare subtype of B-cell precursor (ALL) that could benefit from tyrosine kinase inhibitors (29–31). *EBFI-PDGFRB* gene fusion is reported in <1% of B-ALL, Philadelphia like ALL subtype, which also benefit from tyrosine kinase therapy (26, 32). Deletion of the *PTEN* gene was exclusively detected in T-ALL cases, which is associated with resistance to chemotherapy and early treatment failure (33). Deletion in *NF1* and *SUZ12* in T-ALL are associated with poor response to induction chemotherapy (34). We also observed *NF1* and *SUZ12* deletions in two MRD positive cases of B-ALL suggesting that although rare, these can have implications in treatment outcome.

Gene duplication is a recurring phenomenon in cancer and a major driving force in the gain of biological functions that leads to over-expression and alteration of gene expression patterns. This emerged as a strong correlation in our MRD positive cases which predominantly showed gain of copies of several genes including *LEF1, RUNX1, PAR1, NR3C2, PHF6*, and *CASP8AP2*. Over-expression of *LEF1* has been reported to predict unfavorable outcome and the standard-risk B-ALL patients with high *LEF1* expression can possibly benefit from early treatment modifications and alternative molecular therapies, such as agents targeting the *Wnt* signaling pathway (35). Amplification of *RUNX1* is associated with a poor outcome in childhood ALL (16). Currently, FISH with probes directed to *RUNX1* is the only reliable method of detection of duplication of 21q with amplification of *RUNX1* in ALL. We could reliably detect *RUNX1* amplification in 27% of our cases and thus, dMLPA provides an alternate platform for its detection. Gene duplication of the *PAR1* region has been reported to be associated with *KMT2A-AFF1* cases and a similar profile was also observed in two of our cases (36).

Risk stratification algorithms are regularly revisited for better refinement and monitoring of prognostic factors. Our findings demonstrated the significance of both cytogenetics and CNAs for prognostication. Integration of CNAs with cytogenetic risk stratification allowed identification of subgroups with variable outcomes in the MRD positive high risk group and MRD negative intermediate category because CNAs were detected that are associated with poor prognosis. These intermediate risk group patients can therefore be considered for de-intensification or intensification of chemotherapy. Patients with genetic profiles associated with poor prognosis could be considered for targeted or alternative modalities. MRD status combined with molecular genetic profiling maybe valuable in monitoring and clinical management of these patients. Since chromosomal abnormalities are insufficient to initiate leukemia, the detection of cooperating genetic lesions guide a better risk prognostication, aid in disease monitoring and predict the probability of relapse. However, few limitations remain in this technology such as it is restricted to probe sequence specificity, and silent copy number changes such as inversions or translocations and sequence changes (SNPs, indels) remain undetected. In conclusion, dMLPA is a robust, reliable and a valuable alternative technology for rapid identification of key CNAs in ALL patients, which warrants evaluation in larger cohorts.

## Data Availability

The datasets generated for this study are available on request to the corresponding author.

## Ethics Statement

The studies involving human participants were reviewed and approved by Institute Ethics Committee, All India Institute of Medical Sciences, New Delhi, India. Written informed consent from the participants' legal guardian/next of kin was not required to participate in this study in accordance with the national legislation and the institutional requirements.

## Author Contributions

DT designed and conducted the study, performed the data interpretation, statistical analysis, and wrote the manuscript. GK and RG conceived the idea, performed the data interpretation, statistical analysis, and wrote the manuscript. SS and AB-S are the team from MRC Holland who provided the reagents and assisted with data analysis. IK performed the technical experiments of dMLPA and MLPA. RA compiled the clinical data. LR and VS performed the statistical analysis. PV and SJ performed the RT-PCR experiments. LK, AS, SB, and RS are the clinical faculty who treated the patients during the course of this study.

### Conflict of Interest Statement

The authors declare that the research was conducted in the absence of any commercial or financial relationships that could be construed as a potential conflict of interest.
