# Supplementary material for: Rapid Identification of Key Copy Number Alterations in B- and T-Cell Acute Lymphoblastic Leukemia by Digital Multiplex Ligation-Dependent Probe Amplification
Source: Front Oncol. 2019 Sep 13;9:871. doi: 10.3389/fonc.2019.00871 (PMC6753626; doi:10.3389/fonc.2019.00871)
Supplement: Supplementary file 1 [file Data_Sheet_1.docx]

**SUPPLEMENTARY MATERIAL**

**SUPPLEMENTARY TABLE S1: Frequency distribution of CNAs in B-ALL and T-ALL cases**

|  |  |  | **B-ALL (n=121)** | | **T-ALL (n=27)** | |
| --- | --- | --- | --- | --- | --- | --- |
| **S.No.** | **Gene ID** | **Chr** | **LOSS**  **Cases (%)** | **GAIN**  **Cases (%)** | **LOSS**  **Cases (%)** | **GAIN**  **Cases (%)** |
| **1** | **TAL1** | 1 | 0 | 0 | 5 (18.5%) | 0 |
| **2** | **STIL** | 1 | 0 | 0 | 5 (18.5%) | 0 |
| **3** | **IKZF2** | 2 | 1 (0.8%) | 0 | 0 | 0 |
| **4** | **FHIT** | 3 | 0 | 1 (0.8%) | 0 | 0 |
| **5** | **CD200** | 3 | 5 (4%) | 1 (0.8%) | 0 | 0 |
| **6** | **BTLA** | 3 | 7 (5.8%) | 1 (0.8%) | 0 | 0 |
| **7** | **TBL1XR1** | 3 | 10 (8.3%) | 1 (0.8%) | 1 (~4%) | 0 |
| **8** | **LEF1** | 4 | 2 (1.7%) | 12 (9.9%) | 1 (~4%) | 2 (7.4%) |
| **9** | **NR3C2** | 4 | 3 (2.5%) | 14 (12%) | 0 | 1 (~4%) |
| **10** | **NR3C1** | 5 | 2 (1.7%) | 7 (5.8%) | 0 | 0 |
| **11** | **PDGFRB** | 5 | 2 (1.7%) | 7 (5.8%) | 0 | 0 |
| **12** | **EBF1** | 5 | 5 (4%) | 7 (5.8%) | 1 (~4%) | 0 |
| **13** | **CASP8AP2** | 6 | 6 (5%) | 18 (15%) | 3 (11%) | 2 (7.4%) |
| **14** | **MYB** | 6 | 2 (1.7%) | 19 (16%) | 0 | 3 (11%) |
| **15** | **IKZF1** | 7 | **27 (22%)** | 3 (2.5%) | 2 (7.4%) | 0 |
| **16** | **EPHA1** | 7 | 4 (3.3%) | 3 (2.5%) | 2 (7.4%) | 0 |
| **17** | **EZH2** | 7 | 4 (3.3%) | 4 (3.3%) | 1 (~4%) | 1 (~4%) |
| **18** | **TOX** | 8 | 0 | 10 (8.3%) | 1 (~4%) | 0 |
| **19** | **JAK2** | 9 | 10 (8.3%) | 2 (1.7%) | 4 (~15%) | 0 |
| **20** | **MLLT3** | 9 | 17 (14%) | 1 (0.8%) | 4 (15%) | 0 |
| **21** | **MTAP** | 9 | 17 (14%) | 1 (0.8%) | **9 (33.3%)** | 0 |
| **22** | **CDKN2A** | 9 | **27 (22%)** | 2 (1.7%) | **16 (59%)** | 0 |
| **23** | **CDKN2B** | 9 | **27 (22%)** | 2 (1.7%) | **16 (59%)** | 0 |
| **24** | **PAX5** | 9 | **23 (19%)** | 3 (2.5%) | 3 (11%) | 0 |
| **25** | **ABL1** | 9 | 0 | 2 (1.7%) | 0 | 2 (7.4%) |
| **26** | **NUP214** | 9 | 0 | 2 (1.7%) | 0 | 2 (7.4%) |
| **27** | **NOTCH1** | 9 | 0 | 4 (3.3%) | 0 | 1 (~4%) |
| **28** | **PTEN** | 10 | 0 | **23 (19%)** | 4 (14.8%) | 0 |
| **29** | **ADD3** | 10 | 1 (0.8%) | 8 (6.6%) | 0 | 0 |
| **30** | **LMO1** | 11 | 0 | 4 (3.3%) | 0 | 0 |
| **31** | **LMO2** | 11 | 0 | 5 (4%) | 0 | 0 |
| **32** | **CD44** | 11 | 0 | 1 (0.8%) | 0 | 1 (~4%) |
| **33** | **RAG2** | 11 | 2 (1.7%) | 6 (5%) | 0 | 0 |
| **34** | **ETV6** | 12 | 17 (14%) | 2 (1.7%) | 0 | 0 |
| **35** | **BTG1** | 12 | 8 (6.6%) | 4 (3.3%) | 0 | 0 |
| **36** | **RB1** | 13 | 5 (4%) | 0 | 0 | 0 |
| **37** | **IGHM** | 14 | 11 (9%) | **20 (16.5%)** | 0 | 0 |
| **38** | **SPRED1** | 15 | 0 | 2 (1.7%) | 0 | 0 |
| **39** | **CREBBP** | 16 | 1 (0.8%) | 2 (1.7%) | 0 | 0 |
| **40** | **CTCF** | 16 | 6 (5%) | 1 (0.8%) | 0 | 0 |
| **41** | **TP53** | 17 | 6 (5%) | 21 (17%) | 0 | 0 |
| **42** | **NF1** | 17 | 0 | 18 (15%) | 2 (7.4%) | 0 |
| **43** | **SUZ12** | 17 | 3 (2.5%) | 18 (15%) | 2 (7.4%) | 0 |
| **44** | **IKZF3** | 17 | 1 (0.8%) | 17 14%) | 0 | 1 (~4%) |
| **45** | **PTPN2** | 18 | 0 | 9 (7.4%) | 0 | 0 |
| **46** | **ERG** | 21 | 5 (4%) | **37 (30.6%)** | 0 | 1 (~4%) |
| **47** | **RUNX1** | 21 | 2 (1.7%) | **39 (32.2%)** | 0 | 1 (~4%) |
| **48** | **VPREB1** | 22 | **22 (18%)** | 3 (2.5%) | 0 | 0 |
| **49** | **IGLL1** | 22 | 2 (1.7%) | 0 | 0 | 0 |
| **50** | **PAR1** | X | 2 (1.7%) | **23 (19%)** | 2 (7.4%) | 0 |
| **51** | **CRLF2** | X | 1 (0.8%) | 19 (15.7%) | 2 (7.4%) | 0 |
| **52** | **CSF2RA** | X | 1 (0.8%) | 19 (15.7%) | 2 (7.4%) | 0 |
| **53** | **IL3RA** | X | 1 (0.8%) | 19 (15.7%) | 2 (7.4%) | 0 |
| **54** | **P2RY8** | X | 1 (0.8%) | 19 (15.7%) | 2 (7.4%) | 0 |
| **55** | **DMD** | X | 1 (0.8%) | 16 (13.2%) | 0 | 0 |
| **56** | **PHF6** | X | 0 | **24 (19.8%)** | 1 (~4%) | 1 (~4%) |

**SUPPLEMENTARY TABLE S2: Cases with Hyperdiploidy status (>51 chromosomes) analyzed by dMLPA (n=19)**

| **I.D.** | **Hyperdiploidy (Non-random chromosomal Gain)** | **Karyotyping** | **CNAs**  **Gene Deletions** |
| --- | --- | --- | --- |
| **ID52** | 6, 14, 16, 17, 18, 21, X | 53XY,+X,+6,+13,+14 | *CTCF* |
| **ID53** | 4, 6, 10, 14, 17, 18, 21, X | NA | *CDKN2A/B* |
| **ID54** | 4, 6, 14, 17,18, 21, X | HYPERDIPLOID |  |
| **ID55** | 4, 6, 10, 14, 17, 21, X | NA |  |
| **ID56** | 4, 10, 14, 17, 18, 21, X | FAILED | *ETV6* |
| **ID57** | 1, 4, 6, 10, 14, 17, 18, 21, X | NA |  |
| **ID58** | **8, 10, 18, 19, 21, 22, X** | NA | *IKZF1* |
| **ID59** | +6, 10, 14, 21, XX | NA |  |
| **ID60** | 6, 8, 10, 14, 18, 21, X | NA |  |
| **ID61** | 6, 10, 14, 17, 18, 21, X | NA |  |
| **ID62** | 4, 6, 8, 10, 17, 18, 21, X | FAILED |  |
| **ID63** | 4, 6, 8, 14, 17, 18, 21, X | NA | *TBL1XR1, ERG, VPREB1* |
| **ID64** | 5, 6, 10, 14, 17, 18, 21, X | NA |  |
| **ID65** | 6, 7, 17, 18, 21, X | FAILED | *CDKN2A/B, MTAP* |
| **ID66** | 6, 8, 14, 21, XX | 52XX,+6,+8 |  |
| **ID67** | 4,7,8, 10, 14, 17, 18, 21, X | NA | *CASP8AP2* |
| **ID68** | 10, 14, 17, 21, X | NA |  |
| **ID69** | 4, 6, 14, 17, 18, 21, X | NA |  |
| **ID70** | 6, 10, 21, XX | NA | *RAG2* |

**SUPPLEMENTARY TABLE S3: Concordance between dMLPA and conventional MLPA**

|  | **dMLPA** | **P202+P335**  **(n=61)** | **P327 (n=35)** | **P383 (n=20)** |
| --- | --- | --- | --- | --- |
| **EBF1** | 6 | 6 | - | - |
| **IKZF1** | 17 | 17 | - | - |
| **JAK2** | 4 | 4 | - | - |
| **CDKN2A** | 34 | 24 | - | 8 |
| **CDKN2B** | 24 | 17 | - | 6 |
| **PAX5** | 18 | 18 | - | - |
| **ETV6** | 11 | 11 | - | - |
| **BTG1** | 3 | 3 | - | - |
| **RB1** | 2 | 3 | - | - |
| **IGHM** | 2 | 1 | - | - |
| **PAR1** | 1 | 2 | - | - |
| **CRLF2** | 1 | 2 | - | - |
| **CSF2RA** | 3 | 4 | - | - |
| **IL3RA** | 4 | 6 | - | - |
| **P2RY8** | 2 | 3 | - | - |
| **ERG** | 34 | - | 34 | - |
| **RUNX1** | 35 | - | 34 | - |
| **TAL1** | 4 | - | - | 4 |
| **STIL** | 4 | - | - | 4 |
| **LEF1** | 2 | - | - | 2 |
| **CASP8AP2** | 4 | - | - | 4 |
| **EZH2** | 1 | - | - | 3 |
| **MLLT3** | 5 | - | - | 5 |
| **MTAP** | 7 | - | - | 7 |
| **ABL1** | 2 | - | - | 2 |
| **NUP214** | 2 | - | - | 2 |
| **PTEN** | 1 | - | - | 1 |
| **CD44** | 1 | - | - | 1 |
| **NF1** | 2 | - | - | 2 |
| **SUZ12** | 2 | - | - | 2 |
| **PHF6** | 8 | - | - | 8 |

**
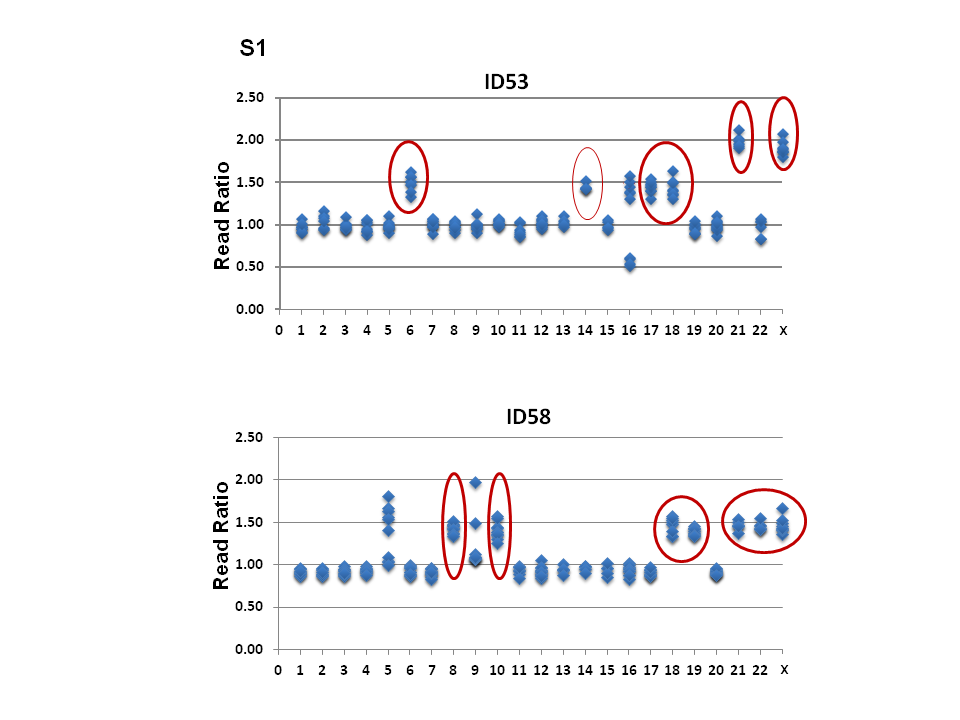
**

**
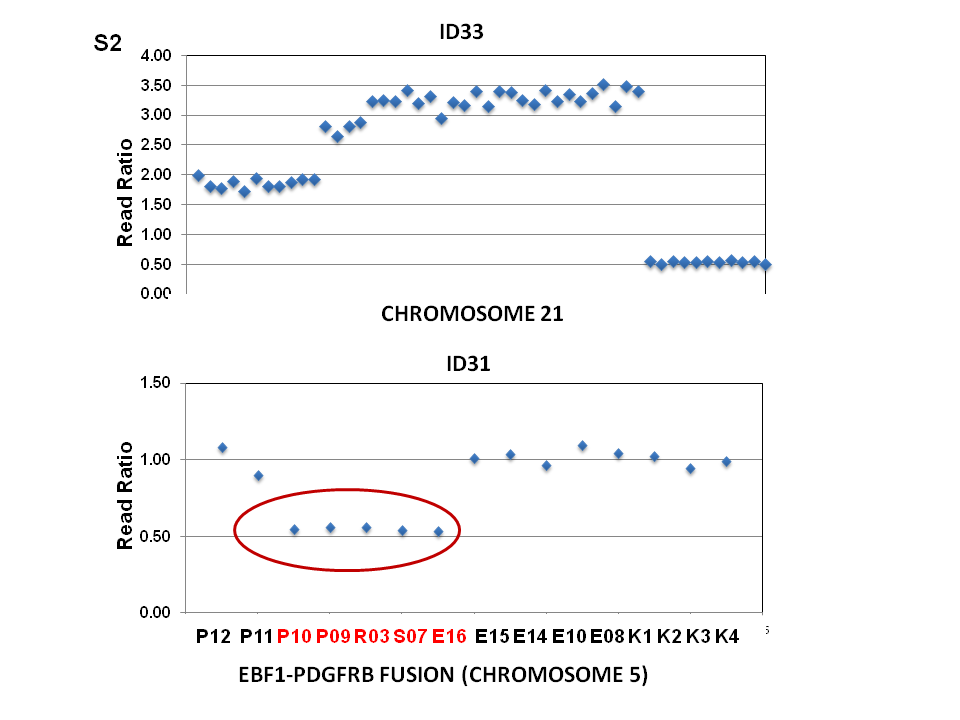
**

**
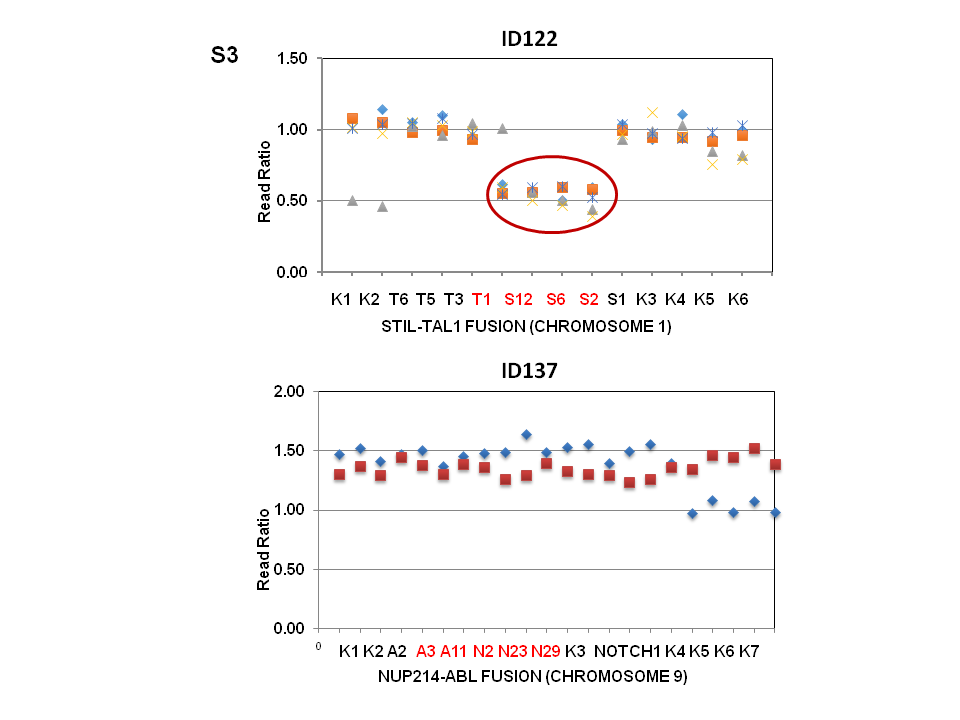
**

**
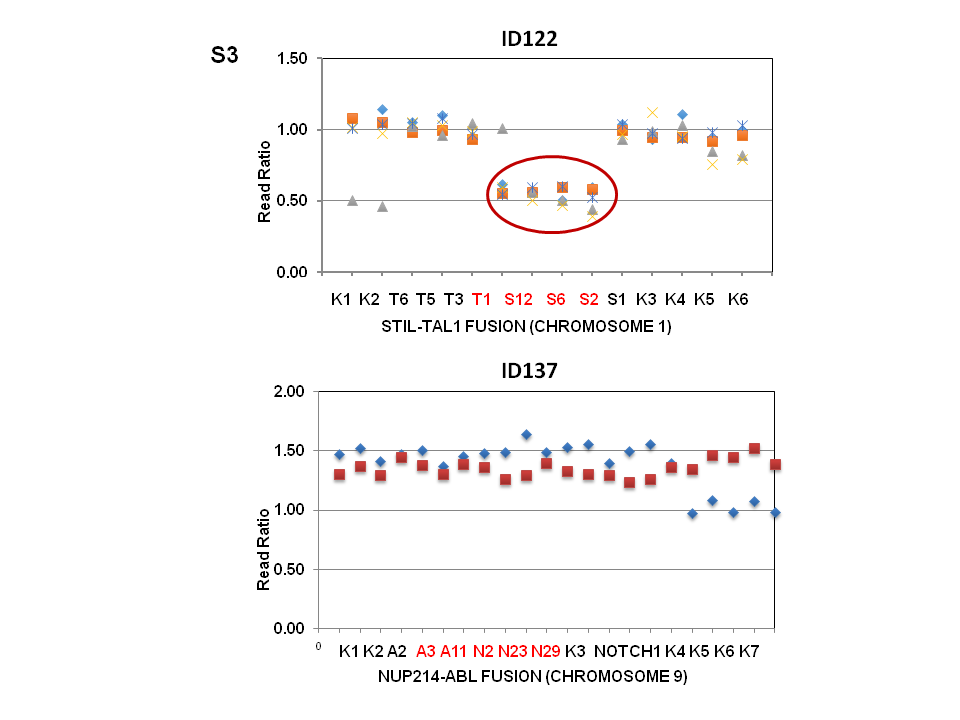
**

**
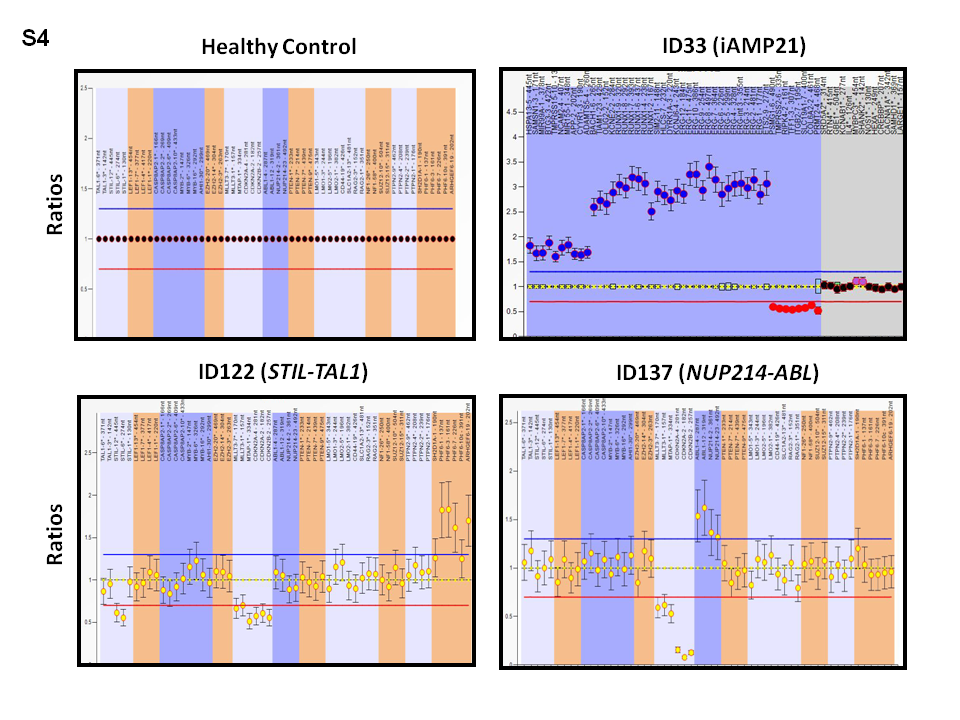
**

**Supplementary Figure Legends:**

**SUPPLEMENTARY FIGURE S1: Hyperdiploidy status identified in B-ALL samples by dMLPA. (A)** A representative B-ALL case showing gain of 1 copy of chromosome 6, 14, 17 and 18 and gain of 2 copies of chromosomes 21 and X highlighted by red circles. **(B)** Gain of single copy of chromosome 8, 10, 18, 19, 21, 22 and X shown in a representative case. The y-axis represents read ratios relative to the reference samples and x-axis shows the chromosome number.

**SUPPLEMENTARY FIGURE S2:** Plot showing iAMP21 amplification in a representative sample (top panel). Intrachromosomal gene fusion EBF1-PDGFRB detected by dMLPA showing read ratios on y-axis and region of exon deletions (bottom panel). Red circle highlights the deleted exons as a result of fusion between Exon 9 of PDGFRB and exon 16 of EBF1. The y-axis represents read ratios relative to the reference samples and x-axis shows the probes represented by each dot.

**SUPPLEMENTARY FIGURE S3:** **(A)** Plot showing intrachromosomal fusion of STIL-TAL1 in 5 cases **(B)** Plot showing NUP214-ABL1 amplification in two cases of T-ALL. Red circle highlights the deleted exons. The y-axis represents read ratios relative to the reference samples and x-axis shows the probes represented by each dot.

**SUPPLEMENTARY FIGURE S4:** **Validation of dMLPA data by conventional MLPA.** Representative plot showing read ratios in a reference sample (Top left panel). Plot showing read ratios of *iAMP21* amplification (Top right panel). *STIL-TAL1* intrachromosomal fusion and deletions in *MLLT3, MTAP and CDKN2A* detected by MLPA P383 probemix (Bottom left panel). ID122 is the same as ID1 in T-ALL heat map (Figure 5). *NUP214-ABL1* amplification detected by standard MLPA (Bottom right panel). ID137 is the same sample as ID16 in T-ALL heat map (Figure 5).
